# Impact of Nano‐Scale Defects on the Macroscopic Amplified Spontaneous Emission in Polycrystalline Perovskite Thin‐Films

**DOI:** 10.1002/adma.202516903

**Published:** 2026-03-26

**Authors:** Chun‐Sheng Jack Wu, E Laine Wong, Hui Li, Jesús Jiménez‐López, Chia‐Kai Lin, Hsu‐Cheng Hsu, Annamaria Petrozza

**Affiliations:** ^1^ Center For Nano Science and Technology Istituto Italiano di Tecnologia Milano Italy; ^2^ Department of Photonics National Cheng Kung University Tainan Taiwan

**Keywords:** amplified spontaneous emission, defect, hyperspectral imaging, laser, perovskite

## Abstract

Perovskite thin film‐based laser diodes have emerged as promising candidates for on‐chip laser sources. However, despite the successful demonstration of optically pumped lasing, the realization of electrically pumped perovskite laser has proved to be quite challenging. Here, we investigated the optical gain mechanism in polycrystalline perovskite thin film by mapping the spatial distributions of amplified spontaneous emission within the heterogeneous thin film and its spatial correlation with the local electronic properties. We discovered that optical gain within these polycrystalline perovskite thin films occurs primarily at defective sites, where despite the low photoluminescence efficiency demonstrated high optical gain efficiency, lower treshold and long photocarrier lifetime. Our findings highlight the importance of defects in the development of electrically pumped laser diodes.

## Graphical abstract

In this work, we discovered that optical gain within these polycrystalline perovskite thin films occurs primarily at defective sites, where, despite the low photoluminescence efficiency, we demonstrated high optical gain efficiency and long photocarrier lifetime. Our findings highlight the importance of defects in the development of electrically pumped laser diodes.

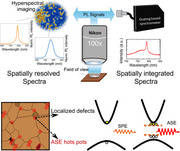

## 1 Introduction

The advent of thin film‐based lasers has led to a paradigm shift in the development of next‐generation coherent sources. In comparison with conventional III‐V semiconductor lasers, thin film‐based lasers can be fabricated on a large variety of substrates such as plastics [1], glass, and silica [2], thus allowing for the production of lightweight, flexible, and scalable laser sources [3, 4]. [5] This flexibility enables the possibility of integration with existing photonic platforms, which in turn leads to the miniaturization and adaptation of thin film laser technology into everyday electronics, wearable electronics [6], and integrated photonic circuits [7].

In the past decades, halide perovskites have shown great potential as an effective gain medium in lasing devices with low threshold optically pumped lasers being demonstrated based on multiple approaches [8, 9, 10, 11, 12] as well as indirect perovskite electrically lasing device has been recently demonstrated [13]. However, despite the initial success demonstrating the achievement of optically pumped lasing in perovskite diodes and indirect electrically driven lasing, developing a direct electrically injected perovskite laser diode has proved to be very challenging. While some reports have demonstrated the achievement of high injected current density above several kA∙cm^−2^ in electrically pumped perovskite diodes [14, 15, 16, 17], there is still no sign of optical amplification. The primary reason is that the threshold carrier density still exceeds the maximum achievable current injection density. Such a bottleneck shows the importance of further optimizing the perovskite gain media for lower thresholds, especially clarifying the optical gain mechanism in perovskite materials under high excitation regimes.

In the pursuit of reducing the threshold in perovskite gain media, an intriguing observation emerged during the early stages of research when perovskite thin film fabrication techniques were still under development. Despite the low quality of these thin films, the reported ASE thresholds were remarkably low at room temperature (RT). i.e. the first report on perovskite ASE by Xing et al. in 2014 demonstrated a threshold of just 10 µJcm^−2^ with MAPbI_3_ thin films [18]. A follow‐up in 2017, Perumal et al. demonstrated a threshold of 9 µJcm^−2^ with a very rough polycrystalline MAPbI_3_ film [19]. Surprisingly, despite a decade of advancements that have significantly improved the quality of MAPbI_3_‐based perovskite thin films, progress in lowering the ASE threshold has been limited. A report by Wen et al. in 2023 showed an RT ASE threshold of 15 µJcm^−2^ with bromide‐doped MAPbI_3_ thin films [17], while Elkhouly et al. reported a much higher RT ASE threshold of 198 µJcm^−2^ by the end of 2024 with a compact MAPbI_3_ thin film [14]. Other recent reports have shown higher ASE thresholds with carefully fabricated MAPbI_3_ thin films [20, 21, 22]. Here, an ASE threshold of 58 µJcm^−2^ was obtained with a state‐of‐the‐art solvent engineering technique [23, 24, 25]. We recently observed that thin films with higher photoluminescence quantum yield (PLQY), optimized for efficient light emitting diodes, exhibited higher optically pumped ASE thresholds than those with lower PLQY, raising a paradox between film quality and ASE threshold [26]. This discrepancy underscores the need for a deeper investigation into the factors influencing ASE thresholds in perovskite gain media, which is important on the path toward the realization of electrically pumped perovskite laser diodes.

In this work, we employed a multimodal microscopy approach to study the optical gain mechanism in perovskite thin films. The photoluminescence (PL) images of the ASE are monitored by fluorescence hyperspectral imaging microscopy [27], particularly in areas where a spatial inhomogeneity in ASE was observed. Then, the correlation between scanning electron microscopy (SEM) image and ASE image shows that ASE originated from localized hot spot regions located at the boundary of the perovskite grains. To further investigate it, the correlation between photoemission electron microscopy (PEEM) spectra and ASE image shows that these localized regions exhibit higher densities of defect states, demonstrating the positive correlation between ASE and the defect states. Finally, the reason ASE emits preferentially from the defective regions is explained using fluorescence lifetime imaging spectroscopy (FLIM). Just below the ASE threshold, the carrier lifetime is found to be prolonged in these defective regions due to carrier trapping in defect states, further confirming the essential defect energy level configuration required to form a three‐level system and sustain population inversion. We further rule out the possibility of enhanced light outcoupling to be the reason behind the concentration of ASE along the grain boundaries by demonstrating ASE onset does not follow the physical fundamental of optical scattering. Also, by demonstrating that passivated sample with larger grains and higher PLQY has a comparatively higher ASE threshold than its pristine counterpart with smaller grains and lower PLQY.

The ASE phenomenon has been considered as a macroscopic quantum effect expected to happen homogeneously as the excitation fluence exceeds the threshold [28, 29]. This can be easily understood since conventional laser gain media, such as single crystals, gases, and epitaxially grown thin films, are usually spatially uniform with the same quantum states. Unlike the traditional laser gain media, the electronic states and excited state population of polycrystalline thin films might differ locally. The inhomogeneous carrier density could result in localized differences in the ASE thresholds, as we observed in this work. This is important on the path of realizing electrically pumped non‐epitaxially grown laser diodes, where the presence of defects is always considered as an undesired species that causes severe joule heating under high current injection [30, 31, 32]. Yet, the observations in this work highlight the necessity of defect states for achieving optical amplification, emphasizing the importance of managing defect density to balance optical gain and charge injection efficiency.

## 2 Results

**FIGURE 1:**
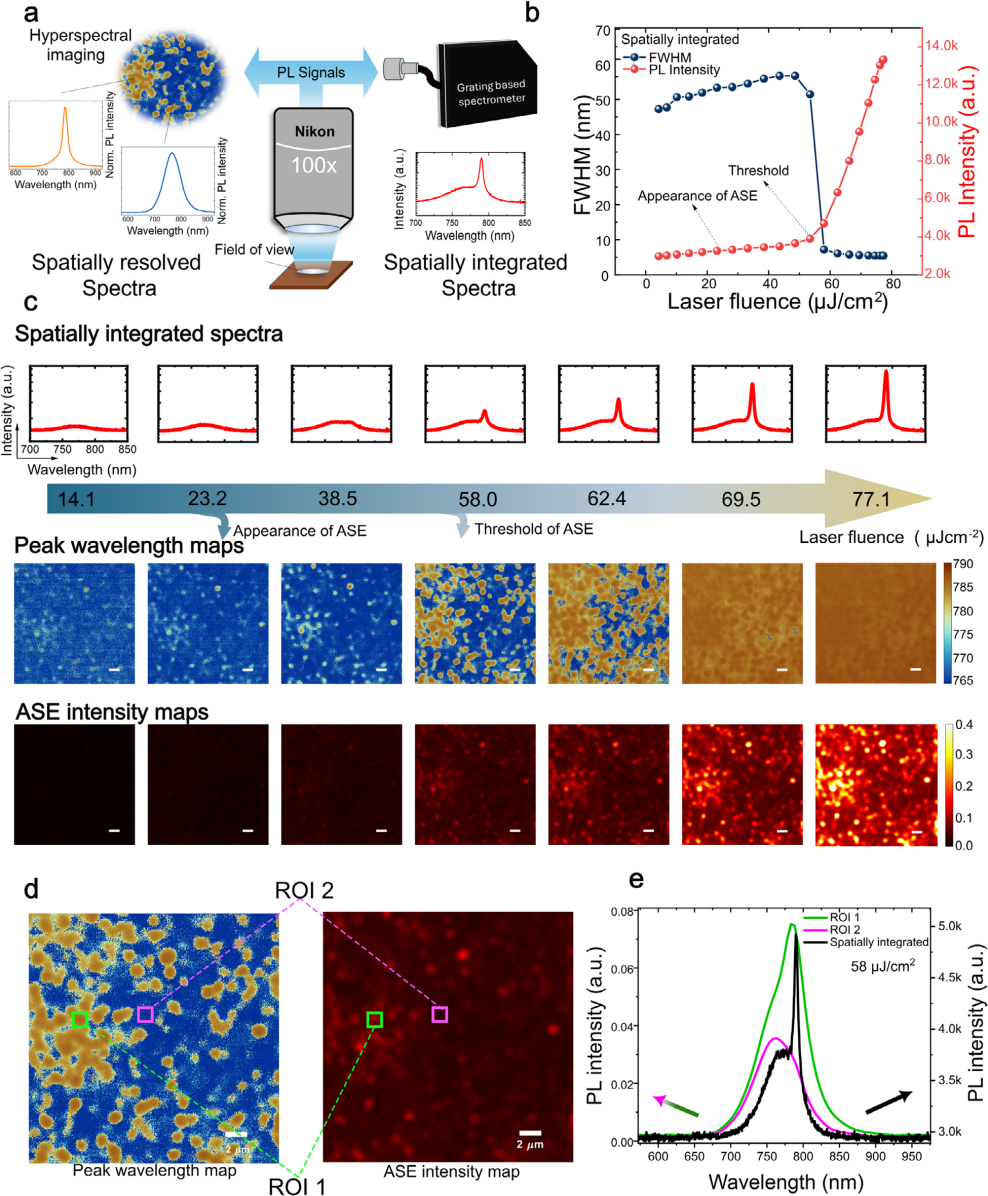
Spatially inhomogeneous ASE in perovskite thin films. (a) The power‐dependent PL measurement setups, the PL signals are collected by both a fiber‐coupled grating‐based spectrometer and a TWINS hyperspectral camera. The grating‐based spectrometer collects the spatially integrated PL signal of the whole field of view, while the hyperspectral camera provides spatially resolved PL spectra. (b) The intensity at 790 nm and FWHM of the spatially integrated PL spectra, as a function of laser fluence, shows the ASE threshold of ≈ 58 µJcm^−2^. (c) Power‐dependent spatially integrated PL spectra and spatially resolved ASE maps. Top row: The spatially integrated spectra captured by the fiber‐coupled grating‐based spectrometer, the *x*‐and *y*‐axis remain the same for each spectrum. Second row: The power‐dependent peak wavelength maps, the *z*‐axis corresponding to the peak emission wavelength of each spot, blue‐765 nm, representing the spontaneous emission wavelength; red‐790 nm, representing the ASE wavelength. Bottom row: the spatially resolved PL intensity map of the ASE wavelength (790 nm), indicating the inhomogeneity of ASE distribution. (d) The spatially resolved maps at 58 µJcm^−2^, left: peak wavelength map, right: ASE intensity map. Green ROI: ASE region, magenta ROI: non‐ASE region. (e) Comparison between spatially integrated spectrum (black) and spatially resolved spectrum. Scale bar = 2 µm for all the figures.

To monitor how ASE happens in perovskites thin film, we employed fluorescence hyperspectral imaging spectroscopy to detect the photoluminescence (PL) intensity distribution maps of the perovskites under different excitation fluence [33, 34]. This allows us to spatially resolve the PL emission and investigate its correlation with the thin film morphology of the polycrystalline perovskite thin films. Figure 1a shows the experimental setup, where a fiber‐coupled grating‐based spectrometer and a Translating‐Wedge‐Based Identical Pulses eNcoding System (TWINS) [27] hyperspectral camera collects separately the spatially integrated and the spatially resolved PL signals from the same field of view. The former provides the averaged spectra of the whole field of view, similar to a conventional macro‐PL measurement; the latter collects both the spectrally resolved PL images and spatially resolved PL spectra.

We first demonstrate the evolution of the ASE in a model system, a polycrystalline MAPbI_3_ thin film as a function of photo‐excitation fluence. Figure 1b presents the PL peak intensity and the full‐width at half‐maximum (FWHM) of the spatially integrated PL spectra of the MAPbI_3_ sample as a function of excitation fluence. We define the macroscopic ASE threshold (58 µJcm^−2^) at the turning point where the PL peak intensity starts to increase nonlinearly and the FWHM starts to narrow. In comparison to the ASE threshold defined from the spatially integrated PL spectra, from the peak wavelength map depicting the PL peak wavelength (second row of Figure 1c, the z‐axis corresponds to the peak emission wavelength, blue‐765 nm, red‐790 nm, representing the spontaneous emission and ASE wavelength, respectively.), the emergence of ASE can already be observed in some regions starting from 23.2 µJcm^−2^, a much lower excitation fluence. The spatial regions showing the appearance of ASE then gradually dominate the whole field of view with increasing excitation fluence. This demonstrates that the threshold estimated from the conventional macro‐PL measurement is only established when ASE dominates the entire field of view. Similarly, from the ASE intensity maps measured at 790 nm (ASE peak wavelength, bottom row of Figure 1c), we observed that the ASE emits preferentially from localized hot‐spots. It is the first time we can microscopically monitor the ASE process in a polycrystalline thin film and thus unveil its evolution with excitation fluence densities. To better exhibit the presence of localized threshold difference, we plotted the peak wavelength map and ASE intensity map at the threshold condition (58 µJcm^−2^) in Figure 1d, the region of interest (ROI) 1 and 2 represent the ASE hot‐spot and no ASE region, respectively. The spectra are shown in Figure 1e, where the black line indicates the spatially integrated spectrum of the whole field of view, showing a clear ASE signal. The green and magenta lines are the spatially resolved spectra of ROI 1 and 2, showing that some areas have achieved ASE but some have not. We further compared the PL maps with a different region of the same thin film in Note S1 and discovered that the density of these ASE hot‐spots could be the leading cause for the differences in the apparent macroscopic ASE threshold. This then begs the question: what are these localized ASE hot‐spots?

**FIGURE 2:**
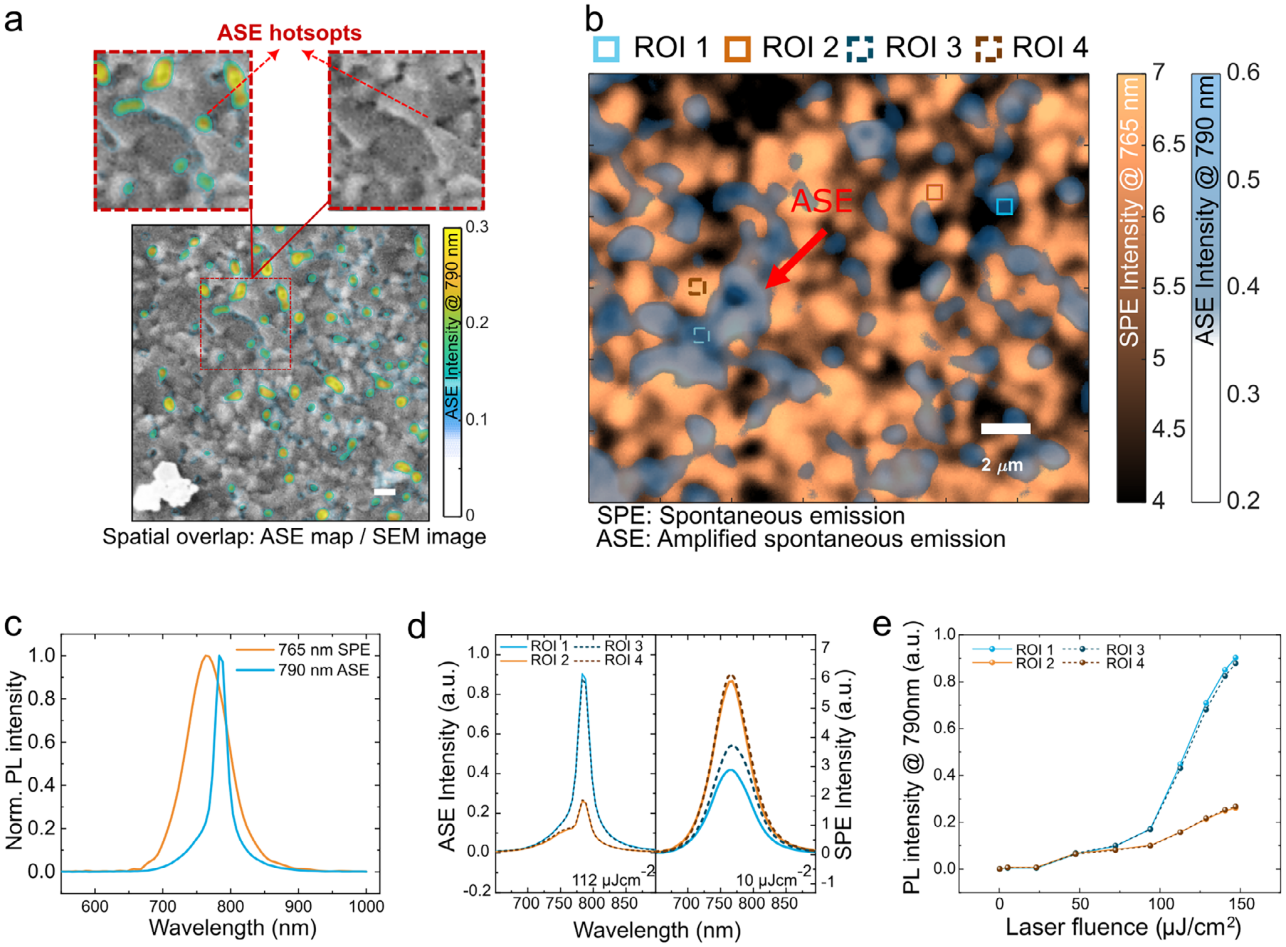
Anti‐correlation emission regions between SPE and ASE maps. (a) Spatial overlap between the SEM and ASE intensity map. The low‐intensity part (blue color) of the ASE image is set to be transparent to demonstrate the data better. The yellow regions represent the intense ASE region. The red inset figures are the zoomed‐in images to show the intense ASE areas that highly correlate with the thin film grain boundaries. (b) spatial overlap between the spontaneous emission (SPE) / ASE intensity maps, Brown: PL intensity maps of 765 nm, representing the spontaneous emission region; Blue: PL intensity maps of 790 nm, representing the ASE hot‐spots. The original images are shown in Figure. S7. ROI 1,3: ASE hot‐spots, ROI 2,4: spontaneous emission hot‐spots, (c) The corresponding spatially integrated spectra of the maps, the spontaneous emission map was measured below the ASE threshold (≈ 10 µJcm^−2^); the ASE map was measured above the threshold (≈ 112 µJcm^−2^). (d) ASE (left) and spontaneous emission (right) spectra of the corresponding ROI. ROI 1 & 3 show a low spontaneous emission intensity but intense ASE intensity, and ROI 2 & 4 show a high spontaneous emission intensity but low ASE intensity. (e) power‐dependent PL intensity of the corresponding ROI. ROI 1 and 3 show a low ASE threshold; ROI 2 and 4 show a high ASE threshold. The high spontaneous emission regions = high ASE threshold regions, and vice versa. Scale bar = 2 µm for all the figures.

To unveil the correlation between these ASE hot‐spots and the thin film morphology, we employed SEM to provide high spatial resolution images of the thin film morphology. To do so, we first deposited gold microparticles onto the perovskite thin films as fiducial markers for the correlative studies between the spatially resolved PL images (Figure. S2) and the SEM images (Figure. S3). Figure 2a shows the SEM image overlapped with the ASE image (790 nm, above threshold, 132 µJcm^−2^) using the fiducial Au marker. The yellow color depicts the ASE regions, while the SEM image is greyscale. The figure shows that these ASE hot‐spots sit at the perovskite grain boundaries. This is unexpected, as one would have expected the grain boundaries to be more defective, leading to lower PL emission and thus higher ASE thresholds.

To further clarify the relation between the grain boundaries and the ASE hot‐spots, we spatially overlap the PL maps below and above the ASE threshold in Figure 2b. The spontaneous emission (SPE) map is the quasi‐steady‐state PL intensity map of 765 nm under a low excitation fluence of 10 µJcm^−2^ (performed under 500 kHz laser repetition rate to reach quasi‐steady‐state condition), where only spontaneous emission exists. The ASE intensity map is the PL map of 790 nm under an excitation fluence of 112 µJcm^−2^ (performed under 2 kHz laser repetition rate to prevent material degradation under high fluence), where ASE dominates the entire field of view. A comparison of the two spatially integrated spectra of Figure 2b is shown in Figure 2c, where different spectral peaks are highlighted, SPE: 765 nm, ASE: 790 nm. The map shows that the ASE hot‐spot areas always follow the edge of the spontaneous emission regions, especially from the highlighted region indicated by the red arrow. The ASE regions mostly coincide with regions showing lower spontaneous emission intensity. Figure 2d illustrates the spectra extracted from the selected ROIs in Figure 2b to show the anti‐correlation between ASE and spontaneous emission spatial distributions. ROI 1 & 3 (Blue) represent the regions with low spontaneous emission intensity but intense ASE; ROI 2 & 4 (Brown) indicate the regions with high spontaneous emission intensity but low ASE. This observation is further supported by power‐dependent relative PLQY (Figure S4) where the areas with intense ASE (ROI 1 & 3) show lower relative PLQY, and the critical points of the power‐dependent PLQY shift toward higher excitation density. This indicates that the ASE hot‐spots coincide with areas with higher defect density [26, 35], whereas the spontaneous emission bright spots (ROI 2 & 4) coincide with areas exhibiting lower defect density regions but very low ASE intensity. The anti‐correlation between the ASE and spontaneous emission spatial distributions rules out enhanced light outcoupling at the grain boundaries to be the main mechanism behind the ASE hot‐spots as the outcoupling efficiency will affect both ASE and spontaneous emission equally. Figure 2e shows the power‐dependent PL curve at 790 nm, it is noted that the regions with intense ASE exhibit a much lower threshold, meaning that these ASE hot‐spots not only emit much intense ASE signal, but also achieve ASE much more easily. Detailed power‐dependent spectra are shown in Figure S5. Such phenomenon could also be found in FA_0.85_Cs_0.15_PbI_x_Br_3‐x_ perovskite thin films (shown in Figure S6), indicating that such phenomenon is a general trend in 3D perovskite thin films.

**FIGURE 3:**
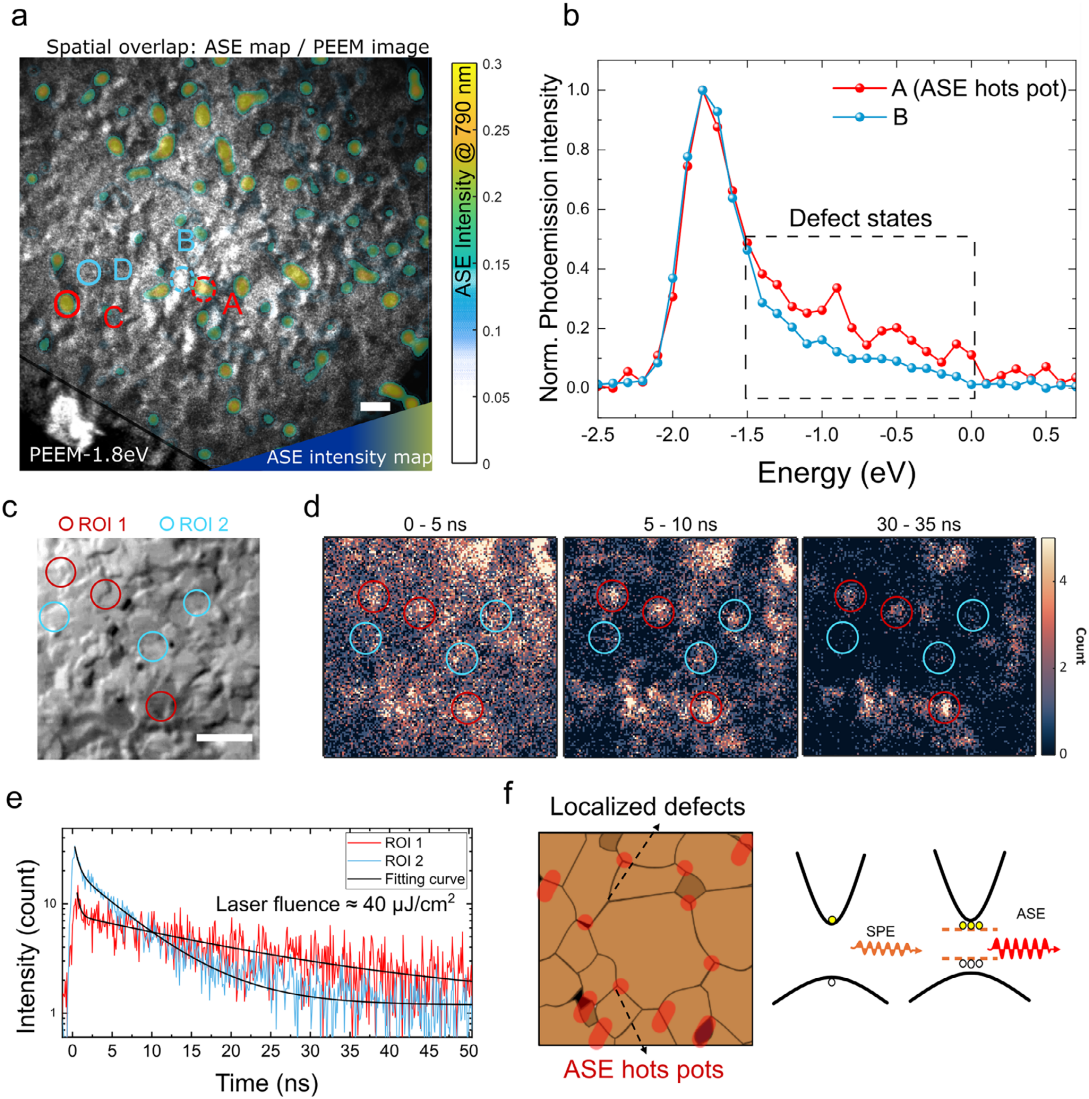
Spatial correlations between ASE hot‐spots, electronic landscape, and carrier dynamics. (a) Spatial overlap between the ASE map and PEEM image. The circle areas represent the selected ROI regions. The red ROIs represent the intense ASE regions; the blue represents weak ASE regions. (b) The PEEM spectra of the corresponding selected areas. The intense ASE regions correspond to regions with higher defect density. (The spectra of C and D are shown in Figure. S9.). (c) The optical image of the MAPbI_3_ thin film. ROI 1 (red) represents the grain boundary regions; ROI 2 (blue) represents well‐grown perovskite grain regions. scale bar = 2 µm. (d) The FLIM images are integrated every 5 ns, normalized to the first interval (image of 0–5 ns). (e) The PL lifetime of the corresponding ROIs. (f) A schematic illustrating the origin of ASE from localized defects of the grain boundaries and the defective states forming the essential carrier accumulation level for achieving population inversion.

To further explore why ASE emits from the grain boundaries, we probe the valence band and inter‐band states locally via PEEM, which allows us to resolve the energy landscape of the thin film surface spatially. In Figure 3a, we overlap the PEEM image (at −1.8 eV binding energy) and the ASE image (790 nm, above threshold, 132 µJcm^−2^). Figure 3b plots the photoemission spectra of the selected ROIs indicated in Figure 3a. We observe that the ROI with higher ASE intensity (red) shows higher photoemission intensity below the valence band edge corresponding to the defect states [33, 36]. We then re‐measured the ASE image after the PEEM measurement, as shown in Figure S8. The ASE hot‐spots remain in the same regions. This demonstrates that the PEEM measurement does not affect the spatial distributions of the ASE.

In our previous work [26], we have demonstrated that shallow defect states aid in the achievement of ASE by creating a reservoir of carriers with prolonged carrier lifetime. To demonstrate if this is the case here, we employed fluorescence lifetime imaging spectroscopy (FLIM) under high excitation fluence (close but below ASE threshold ≈ 40 µJcm^−2^) to compare the PL lifetime difference between the bulk grains and the grain boundaries.

Figure 3c shows the optical image of the film surface, the red and blue rectangles correspond to the selected areas. Figure 3d indicates the FLIM images integrated every 5 ns, normalized to the 0–5 ns image to show the lifetime differences. ROI 1 (red) represents the defective region in the grain boundaries, and ROI 2 (blue) represents the well‐grown perovskite grain. The time‐resolved PL intensity of ROI 1 and 2 is shown in Figure 3e. From the PL dynamics, the well‐grown region exhibits a higher PL intensity but a shorter PL lifetime characterized by a single exponential decay, which could be attributed to the dominance of bimolecular recombination, where free electrons and holes efficiently recombine without significant trapping. In comparison, the defective region shows a lower PL intensity, with a decay profile that features a fast initial component followed by a long tail. This suggests that radiative recombination in the defective region involves both a rapid monomolecular trap‐assisted process and prolonged carrier trapping by shallow trap states [37].

Together, the FLIM images and the PL lifetime dynamic plot confirm our hypothesis that the defect states in the grain boundaries could further trap the carriers, leading to a longer lifetime and making it easier to achieve population inversion. The low defect perovskite grains lead to effective radiative recombination, which shows higher spontaneous emission intensity but runs in the opposite direction of the principle of optical amplification. Figure 3f presents a model demonstrating the spatial origin of ASE in 3D perovskites. The three‐level system consists of the conduction and valence band edges together with the shallow defect states. Carriers are captured at these shallow defect states, where they experience an extended lifetime compared to free carriers in the band states. These trapped carriers effectively act as a reservoir, enabling population accumulation and facilitating the optical gain process under strong excitation.

**FIGURE 4:**
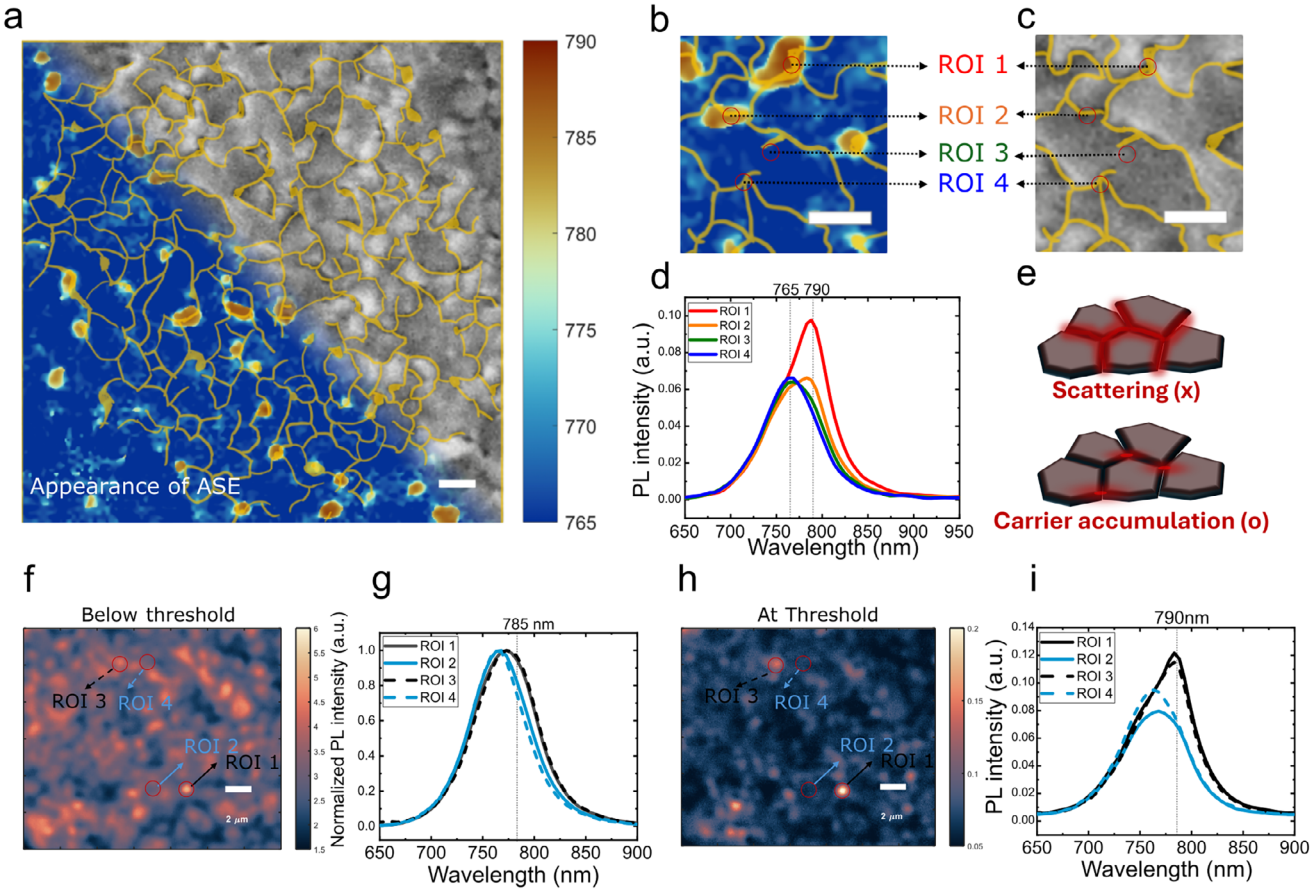
Discussion of lateral light scattering effect of ASE hot spots. (a) Spatial overlap between the SEM and ASE peak wavelength map. The ASE peak wavelength map is plotted at the fluence of first appearance of ASE (≈ 25 µJcm^−2^). The SEM is measured at the exact same region. The yellow lines highlight the grain boundaries. (b,c) the zoom image of Figure 4a, b. ASE peak wavelength map and c. SEM image, respectively. (d) localized emission spectra of corresponding ROIs in Figure 4b,d. (e) the emission profile scheme in the case of light scattering and carrier accumulation. (f) Quasi‐steady‐state PL hyperspectral mapping with laser fluence below threshold (≈ 9 µJcm^−2^). plotted at 785 nm, the red side shoulder of the spontaneous emission. (g) The corresponding localized spectra of the ROIs shown in f, ROI 1 & 3 show a broadening of spontaneous emission, but not ROI 2 & 4. (h) PL hyperspectral mapping with excitation around the threshold fluence (≈ 70 µJcm^−2^), plotted at 785 nm, where the ASE emission peaks are. (i) The corresponding localized spectra of the ROIs shown in (c), ROI 1 & 3 show ASE spectra, but not ROI 2 & 4. Scale bar = 2 µm for all the figures.

One may question whether the ASE hotspot originates from out‐of‐plane scattering at grain boundaries during lateral ASE propagation. To clarify this, we performed a comprehensive analysis that rules out this possibility. Power‐dependent ASE maps were examined and plotted together with the peak emission maps, following the same method used in the second row of Figure 1c. At the excitation fluence corresponding to the onset of ASE (at threshold, ≈ 25 µJcm^−2^), these maps were overlapped with the corresponding SEM morphology image (Figure 4a) to reveal how localized ASE emission correlates with the film morphology. Upon magnifying a single grain, as shown in Figure 4b,c, ASE is observed only in specific regions. The spectra corresponding to selected regions of interest (ROIs) are presented in Figure 4d, where ROI 1 and ROI 2 correspond to ASE hotspots, ROI 3 lies at the center of a grain, and ROI 4 is located at a grain boundary yet shows no ASE signal. This observation contradicts the physical expectations of optical scattering. If ASE hotspot were caused by the out‐of‐plane light scattering, the emission would scatter out of the grains and propagate along the grain boundaries once the threshold was reached, as illustrated schematically in the upper panel of Figure 4e. In such a case, all grain boundaries would exhibit emission around 790 nm. Instead, the ASE displays a localized hotspot‐like pattern, as shown in Figure 4e. This clearly rule out the ASE hot spot emission is originated from out‐of‐plane light scattering effect. We also performed additional experiments to rule out the possibility of random lasing, detail shows in Note S2.

To further substantiate that the ASE emission originates from shallow defect states, we performed quasi‐steady‐state hyperspectral imaging at fluences well below the ASE threshold (≈ 9 µJcm^−2^) to visualize the localized spontaneous emission spectra. We then examined the same region under excitation fluence density above the threshold (≈ 70 µJcm^−2^) to compare the corresponding localized ASE region. From the quasi‐steady‐state PL map, certain regions exhibited a slight redshift and spectral broadening of the spontaneous emission peak. As shown in Figure 4f (emission peak at 765 nm, mapped at 785 nm), several hotspots display enhanced emission intensity at the shoulder of the spontaneous emission peak. The corresponding localized spectra for each ROI are presented in Figure 4g: ROI 1 & 3 show a slight redshift and broadening, whereas ROI 2 & 4 exhibit narrow spontaneous emission peaks.

When the same region is excited with fluences just above the ASE threshold, the ASE emission map Figure 4h reveals that ASE hotspots are strongly correlated with the regions displaying broader spontaneous emission (ROI 1 & 3). The corresponding localized spectra are shown in Figure 4i where ROI 1 & 3 have already reached ASE, while ROI 2 & 4 remain below threshold. These findings confirm that ASE hotspots coincide with areas of increased local lattice disorder and higher shallow defect density, leading to broader spontaneous emission and carrier accumulation in these regions.

From the spatial overlap between the SEM images and the ASE image, the ASE has been shown to concentrate at specific hot spots. According to the localized PEEM spectra, ASE areas exhibit a higher photoemission intensity at the sub‐gap states in PEEM spectra, which refers to the defect states [36, 38]. Finally, the FLIM measurement shows that the carrier lifetime was prolonged in the defective regions, forming the essential carrier accumulation states for achieving population inversion. Based on the results mentioned above, it appears that the defect states at the perovskite grain boundaries are crucial for the achievement of low threshold ASE.

**FIGURE 5:**
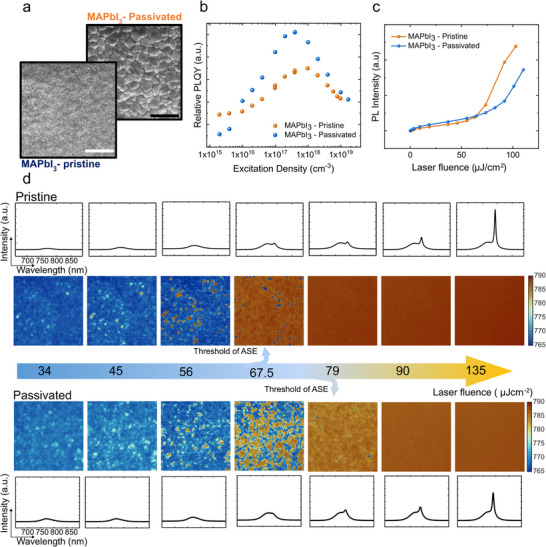
ASE threshold controlled by defect density. (a) SEM images of pristine and passivated MAPbI_3_ thin films. The passivated sample shows a larger grain size compared to the pristine sample. Scale bar = 2 µm. (b) relative PLQY as a function of excitation density. The passivated sample shows a higher PLQY, indicating a reduction in defect. (c) PL intensity as a function of laser fluence. The passivated sample shows a higher ASE threshold. (d) power‐dependent peak wavelength maps and spatially integrated spectra, showing the ASE dominates the whole field of view at a lower excitation fluence density in the pristine sample. The *x*‐and *y*‐axis remain the same for each spectrum.

To further bolster our hypothesis that the ASE originates from the trapped carriers at the defect states, we fabricate a MAPbI_3_ sample with urea as additives to help passivate the defects in the MAPbI_3_ sample [39, 40]. In Figure 5a, we showed the SEM images of pristine and passivated samples, we find that the passivated sample shows a much larger grain size compared to the pristine sample. It is known that the defect density could be reduced by enlarging the grain size [41, 42]. From Figure 5b, the passivated sample shows a higher relative PLQY, indicating a reduced defect density. The ASE threshold comparison is shown in Figure 5c, where the passivated sample shows a higher ASE threshold. This further excludes enhanced light outcoupling at the grain boundaries to be the reason behind the ASE hot‐spots as the larger grain size would lead to longer propagation length, and in turn higher ASE intensity.

Furthermore, the ASE images in Figure 5d show that the ASE dominates the field of view at lower excitation fluence density in the pristine sample than in the passivated sample. This could also be well explained with the ASE intensity maps shown in Figure S10, where the density of the ASE hot‐spots in the pristine sample is much higher than in the passivated sample. Therefore, the ASE dominates the field of view under a lower excitation fluence density. These results further confirm our hypothesis that the defective states in the perovskite thin films are crucial for the achievement of ASE and could explain why the early reports on perovskite ASE show a remarkably low threshold with low‐quality thin films.

## 3 Discussion

Although we realized the importance of defects in the optical amplification of polycrystalline perovskite thin films, carrier trapping during electrical injection often leads to significant Joule heating, posing a fundamental challenge to achieving direct electrically pumped lasing. The main question now is how to achieve high optical gain and good electrical injections simultaneously. Here, we propose a possible strategy to address this paradox.

3.1

Intrinsic defects in perovskites can be broadly categorized into shallow and deep defects. Shallow defects, located near the band edges, serve as beneficial carrier reservoirs facilitating population inversion under high excitation, whereas deep defects, typically situated mid‐gap, have a deleterious impact on device performance at low excitation densities but become saturated at high excitation levels [43]. Importantly, deep defects contribute significantly to heat generation and operational instability during electrical injection [44, 45]. Thus, developing selective passivation strategies that effectively suppress deep defects while retaining shallow defects could enable high optical gain with reduced Joule heating under electrical pumping.

## 4 Conclusion

In this work, we showed that the ASE in polycrystalline perovskite thin films have an inhomogeneous spatial distribution, The spatially resolved PL maps revealed that the threshold estimated from conventional macro‐PL measurements is reached only when ASE dominates the entire field of view. Then, the spatial overlap between the SEM images and the ASE maps shows that ASE emits from the grain boundaries. From the overlap of the spontaneous emission and ASE maps, ASE also emits from the defective region with lower spontaneous emission. This was further verified by the correlation between the ASE map and localized PEEM spectra, where ASE emitted from a more defective region with the presence of inter‐band states. Then, we discovered that grain boundaries exhibit a longer PL lifetime compared to well‐grown grains from FLIM spectroscopy results. We therefore deduced that ASE is aided by the local defect at the grain boundary due to carrier accumulation in the defect states.

Finally, we control the defect density of the MAPbI_3_ thin film samples by conventional defect passivation methods, proving that the ASE threshold is highly associated with the density of the defective regions. We have established that to achieve optical gain in perovskites, the presence of defect states is essential to form the three‐level system necessary for population inversion. On the other hand, the presence of high defect density will cause severe joule heating during high current injections, which in turn degrades the perovskite. This work highlights the importance of defect engineering in thin films and finding a balance between low threshold and high joule heating.

## 5 Methods

### 5.1 Sample Preparation

A 1.5 m CH_3_NH_3_PbI_3_ (MAPbI_3_) perovskite precursor solution was prepared by dissolving 1.5 m lead iodide (PbI_2_, TCI, 99.99%), 1.5 m methylammonium iodide (MAI, Greatcell), and 0.3 m methylamine hydrochloride (MACl, Greatcell) in a mixed solvent of N,N‐dimethylformamide (DMF, Sigma–Aldrich) and dimethyl sulfoxide (DMSO, Sigma–Aldrich) at a 9:1 volume ratio. The solution was stirred at 45°C until fully dissolved, then filtered using a 0.22 µm PTFE filter. Next, 60 µL of the precursor solution was dispensed onto the substrates and spin‐coated at 1000 rpm for 10 s, followed by 5000 rpm for 30 s. During the last 10 s of the second spin‐coating step, 500 µL of diethyl ether was dripped onto the spinning substrates. Finally, the films underwent the same two‐step annealing: the substrates were left unannealed for 2 min, followed by annealing at 100°C for 15 min. For samples in Figure 5, the additive for the passivated samples was changed to 10 wt.% Urea, to ensure the observed effect is independent to the chemical additive. The pristine sample was prepared without any additives.

### 5.2 Measurements and Characterizations

All the optical measurements were performed under N_2_ conditions at room temperature, preventing oxygen contamination for the following electron spectroscopy. All the PL measurements were pumped by a femtosecond laser (1030 nm, pulse width ≈ 300 fs, Pharos, Light Conversion) with an external harmonic generator (Hiro, Light Conversion) to generate a second harmonic 515 nm pump. The beam is then collimated into a fiber collimator and sent to a Nikon LV100ND upright microscope focused by a 100x objective. An Ocean Optics Maya2000 Pro grating‐based spectrometer collected the spatially integrated spectra, and a Nireos HERA VIS‐NIR hyperspectral camera collected the spatially resolved PL maps.

The photoemission electron microscopy (PEEM) images and spectra were acquired using a 6 eV ultrafast pulse at a 500 kHz repetition rate. The field of view was set to 30 µm, and the energy resolution of the photoemission spectra was approximately 250 meV. The spectra were referenced to the Fermi edge of nearby gold markers.

The overlap between PL map and electron microscopy measurements was achieved by first performing hyperspectral imaging microscopy in an N_2_‐filled microscope cryostat. After the optical measurements, the sample was transferred back to the glovebox and transported to the PEEM chamber using an N_2_‐filled transfer tube. Finally, SEM measurements were conducted. Precise spatial alignment between the images was ensured by identifying gold markers deposited on the samples.

FLIM imaging utilized a South‐Port JadeMat‐PV inspection system featuring a 405 nm pulse laser (40 MHz), PMT, TCSPC counting system (multi‐Harp 150, Pico Quant) and Galvo‐based scanner. Imaging was performed using a 100x objective with a numerical aperture of 0.8, and the confocal pinhole was set to a size of 50 µm.

## Funding

European Union's Horizon 2020 research and innovation program MSCA‐ITN PERSEPHONe under Grant Agreement No. 956270, ERC project SOPHY under Grant Agreement No. 771528, National Science and Technology Council (NSTC), Taiwan. Grant Agreement No. 111‐2112‐M‐006‐023‐MY3, 113‐2221‐E‐006‐104‐MY3.

## Conflicts of Interest

The authors declare no conflicts of interest.

## Supporting information




**Supporting File**: adma72825‐sup‐0001‐SuppMat.pdf.

## Data Availability

The data that support the findings of this study are available from the corresponding author upon reasonable request.
