# Supplementary material for: Impact of Nano‐Scale Defects on the Macroscopic Amplified Spontaneous Emission in Polycrystalline Perovskite Thin‐Films
Source: Adv Mater. 2026 Mar 26;38(24):e16903. doi: 10.1002/adma.202516903 (PMC13113229; doi:10.1002/adma.202516903)
Supplement: Supplementary file 1 — Supporting File: adma72825‐sup‐0001‐SuppMat.pdf. [file ADMA-38-e16903-s001.pdf]

Supporting Information

Impact of Nano-scale Defects on the Macroscopic Amplified Spontaneous Emission in Polycrystalline Perovskite Thin-films

Chun-Sheng Jack Wu [1], E Laine Wong [1], Hui Li [1], Jesús Jiménez-López [1], Chia-Kai Lin [2], Hsu-Cheng Hsu [2], Annamaria Petrozza [1]**

Chun-Sheng Jack Wu, E Laine Wong, Hui Li, Jesús Jiménez-López, Annamaria Petrozza.
Center for Nano Science and Technology, Istituto Italiano di Tecnologia; via Rubattino 81, Milano, 20134, Italy.
E-mail: Corresponding author: Annamaria.Petrozza@iit.it , E.wong@iit.it

Chia-Kai Lin, Hsu-Cheng Hsu
Department of Photonics, National Cheng Kung University, Tainan, Taiwan.

Supplementary Text

Note 1.

The intensity at 790 nm and FWHM of the averaged PL spectra as a function of excitation density, is shown for different regions of the same $MAPbI_3$ thin film. Region A exhibits a high ASE threshold (≈ 69 μJcm^{-2}), while region B shows a low ASE threshold (≈ 58 μJcm^{-2}). From the peak wavelength maps shown in **Fig S1a**. and **b.** we observed that the ASE signal in the averaged PL spectra can only be detected when the ASE dominates the whole field of view. Although these two regions show different threshold value, we still observe the first appearance of ASE under the same excitation density, as shown in the HSI maps of 23.2 μJcm^{-2} and **Fig S1c**. This makes sense as we are measuring the same sample, the intrinsic threshold of achieving ASE should be the same. The main reason of the differences in threshold is because the density of ASE emitting region of a polycrystalline thin film are different in different regions, as demonstrated by the PL intensity maps of ASE wavelength shown in **Fig S1d.** and **e.** The density of ASE emission hot spots in region B are higher than region A. It is therefore easier to observe ASE in region B.

Note 2. Excluding random lasing

To further rule out the possibility of random lasing, as the hyperspectral camera does not provide enough spectral resolution, we first measured the emission spectra using a high-spectral-resolution grating-based spectrometer (Andor Shamrock 303i, grating: 1200 grooves/mm). **Figure. Note2-1.** Shows high-resolution spectra of two samples, the top one is the amplified spontaneous emission spectrum of $MAPbI_3$ in this work, and the bottom panel is the random lasing spectrum of $FAPbI_3$ developed from a separate context. The spectrum of ASE shows a single broad emission peak, while random lasing shows a chaotic multi-mode peak.

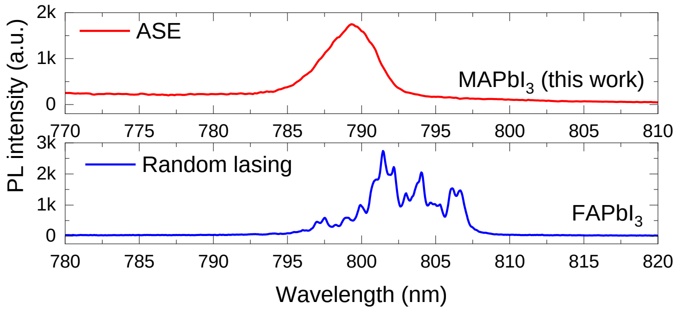

Figure. Note2-1. Spectra comparison between the $MAPbI_3$ ASE sample (top) in this work and the random lasing $FAPbI_3$ sample (bottom) from a separate on-going work of ours.

Next, we performed the hyperspectral imaging of both samples to demonstrate the difference in the emission profile. **Figure. Note2-2** shows the experiment scheme, where we performed two measurements with the excitation area slightly moved. The results are shown in **Figure. Note2-3** and **Figure. Note2-4,** which demonstrates the emission profile of $MAPbI_3$ thin film in this work, and $FAPbI_3$ of a separate on-going work of ours, respectively.

In **Figure. Note2-3a,** the black and red line corresponds to the average spectra of position 1 and 2, which shows identical line shape. In **Figure. Note2-3 b** shows the intensity mapping of ASE wavelength, the top row is position 1, the bottom row is position 2. We can observe that although the excitation position changed, the emission hotspots remain unchanged. The black rectangular boxes identify the same area of the film.

On the other hand, In **Figure. Note2-4a,** the emission spectra changed massively when exciting from different positions, from the emission mapping shown in In **Figure. Note2-4b,** the high-intensity hot spots are randomly distributed with different excitation positions. This is a result of the randomness of the scattering path in the random lasing phenomenon, the bright spots represent the scattering spot which forms the round loop, when the excitation position is changed, even slightly, the scattering path would change.

From the spatial distribution of the emission hotspots of random lasing and ASE, we can rule out random lasing to be the observed phenomenon in this work.

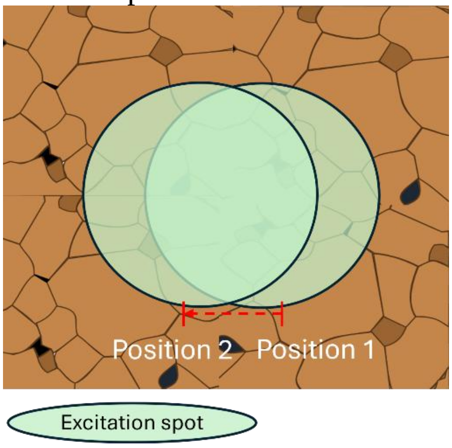

Figure. Note2-2. The experimental scheme, position 2, is slightly moved from position1.

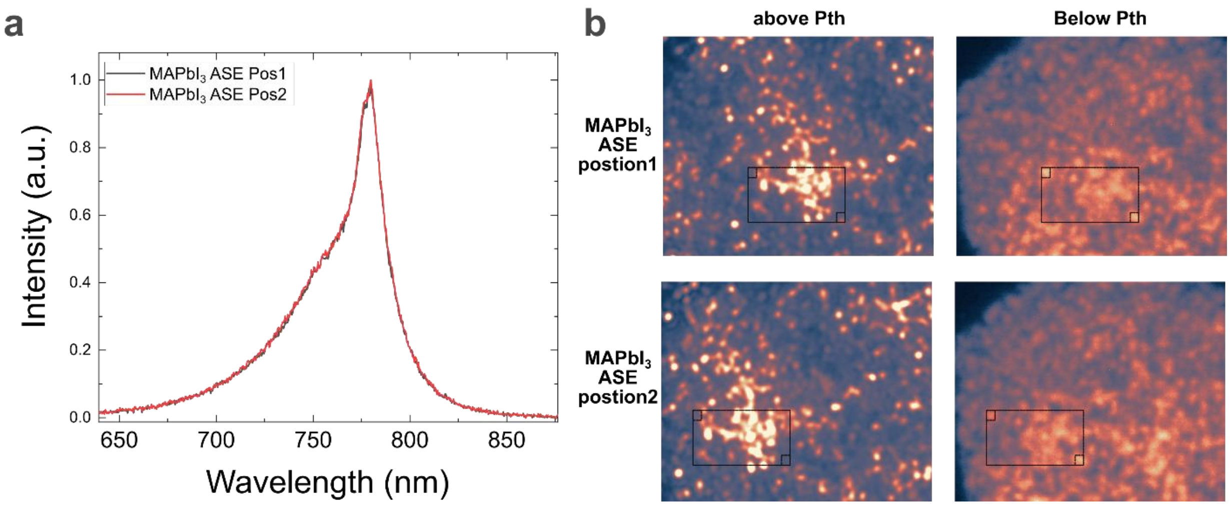

Figure. Note2-3. MAPbI$_3$ ASE characteristics with different excitation positions. a. averaged spectra of excitation position 1 and 2, **b.** the emission map of MAPbI$_3$ thin film, top row: position 1, bottom row: position 2; first column: emission map of 790 nm (ASE peak wavelength), measured above threshold. Second column: emission map of 765 nm (peak wavelength of spontaneous emission). measured below threshold.

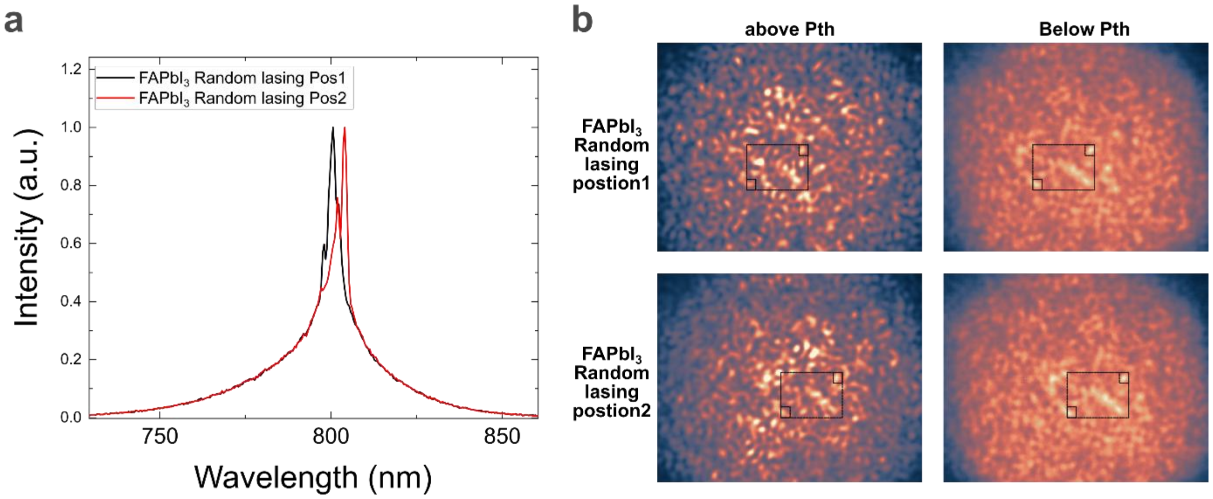

Figure. Note2-4. FAPbI$_3$ random lasing emission characteristics with different excitation positions. a. averaged spectra of excitation position 1 and 2, **b.** the emission map of FAPbI$_3$ thin film, top row: position 1, bottom row: position 2; first column: emission map of 800 nm, measured above threshold. Second column: emission map of 800 nm, measured below threshold.

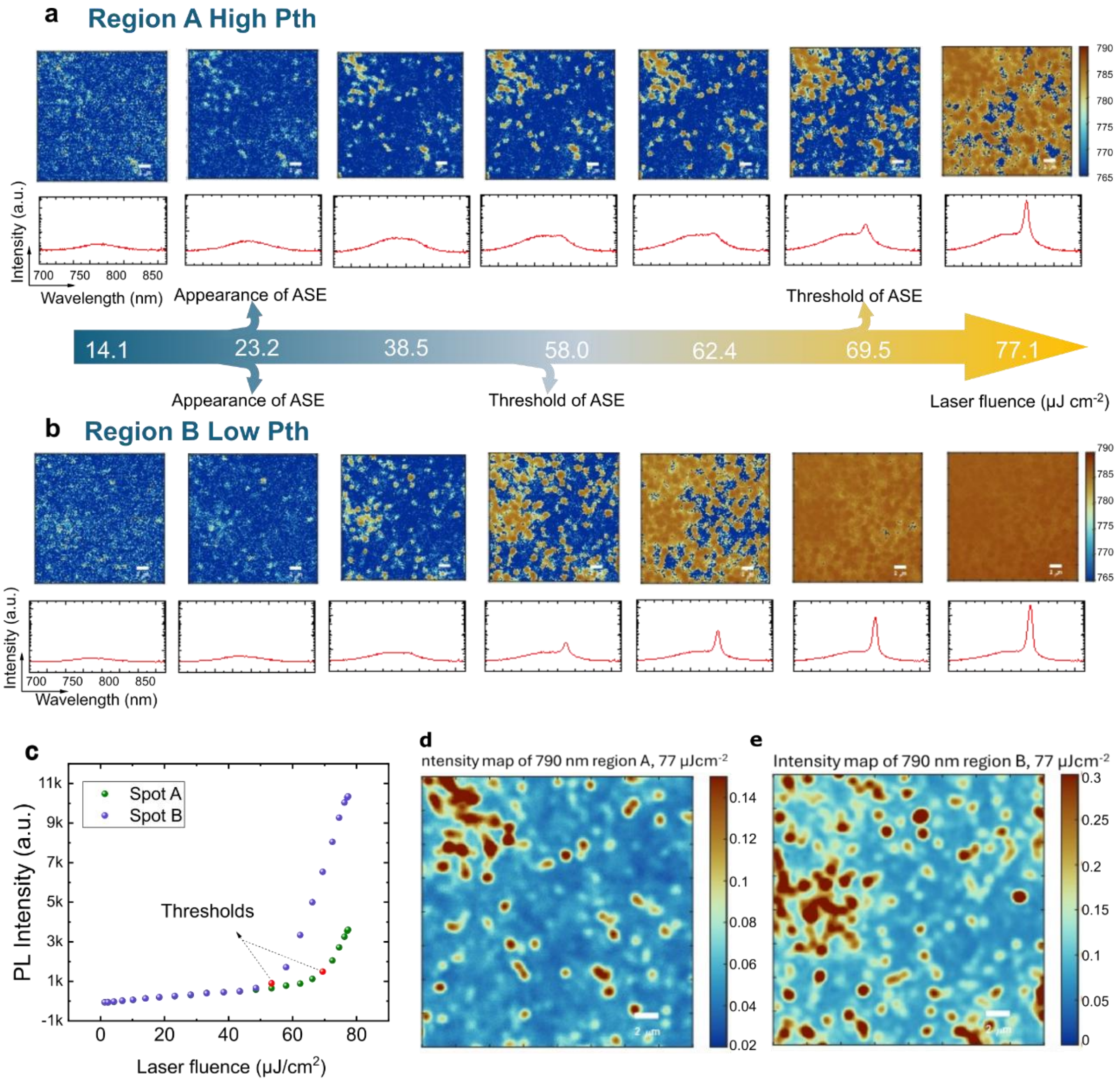

Figure. S1. Threshold comparison within the same sample. Region A exhibits a high ASE threshold (≈ 69 μJcm^{-2}), while region B shows a low ASE threshold (≈ 58 μJcm^{-2}). **a. b.** excitation density-dependent HSI images of region A/B, respectively. ASE threshold is estimated differently due to ASE dominates the field of view under different laser fluence. **c.** PL intensity taken by averaged spectra as a function of excitation density. Although region A and B show different threshold values, the first appearance of ASE is observed under same laser fluence. **d. e.** PL intensity maps of region A and B, respectively. Region B shows a higher density of ASE hot spots, resulting in a dominance under lower excitation density.

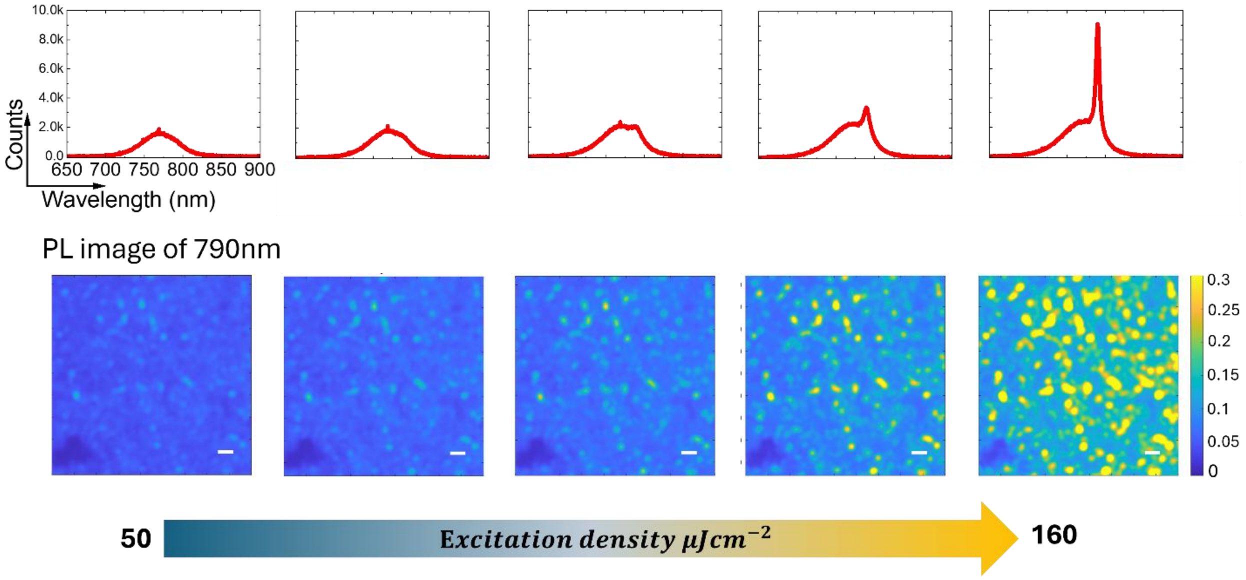

Figure. S2. Power-dependent PL hyperspectral imaging measurement with the gold markers.

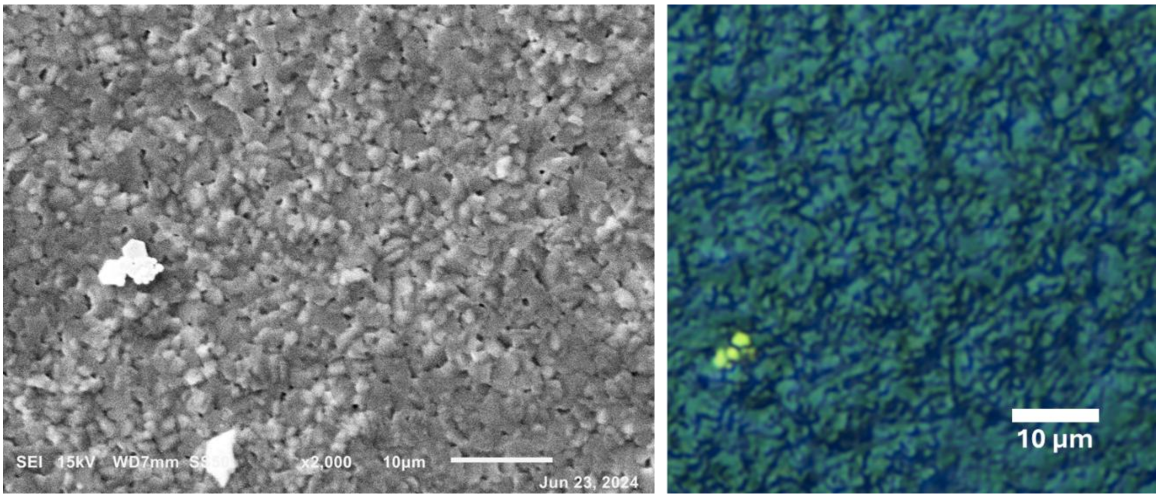

Figure. S3.The SEM and optical image of the gold particle marked region.

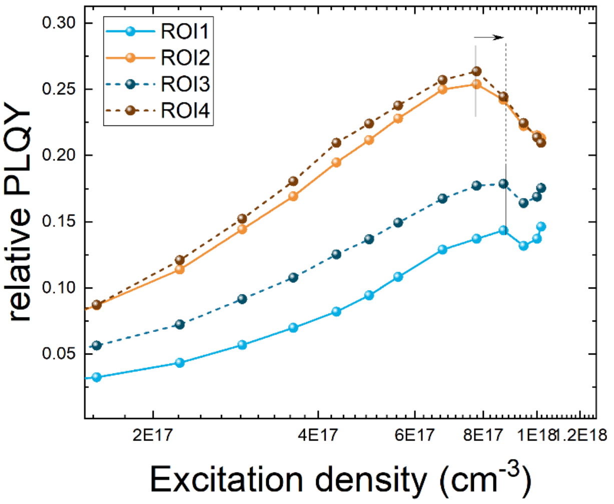

Figure. S4.The power-dependent relative PLQY. The area with intense ASE (ROI 1 & 3) matches with lower PLQY, and the critical point of the power-dependent PLQY shifts towards the higher excitation density.

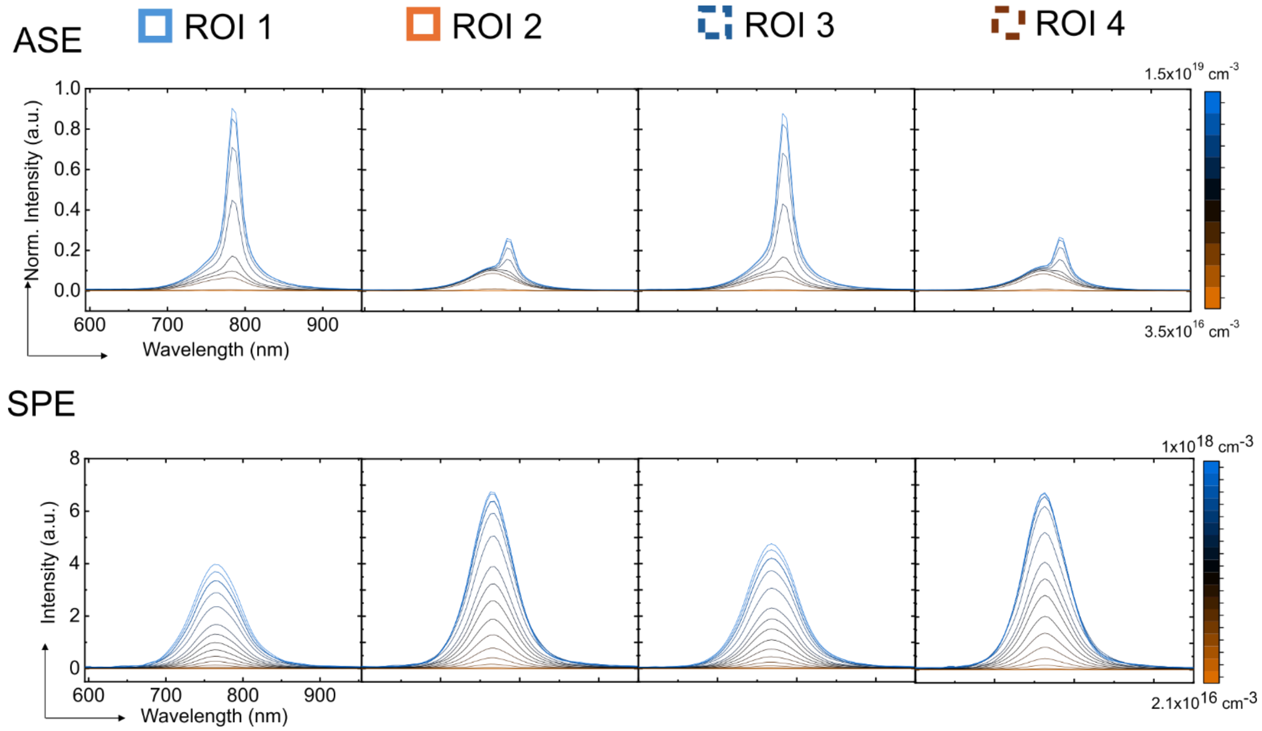

Figure. S5. Localized power-dependent spectra of each ROI. The top row corresponds to the ASE high excitation density regime. The bottom row is the PLQY low excitation density regime. Each ROIs corresponding to the ROIs shown in figure. 2b, each measurement has four ROI with exact positions.

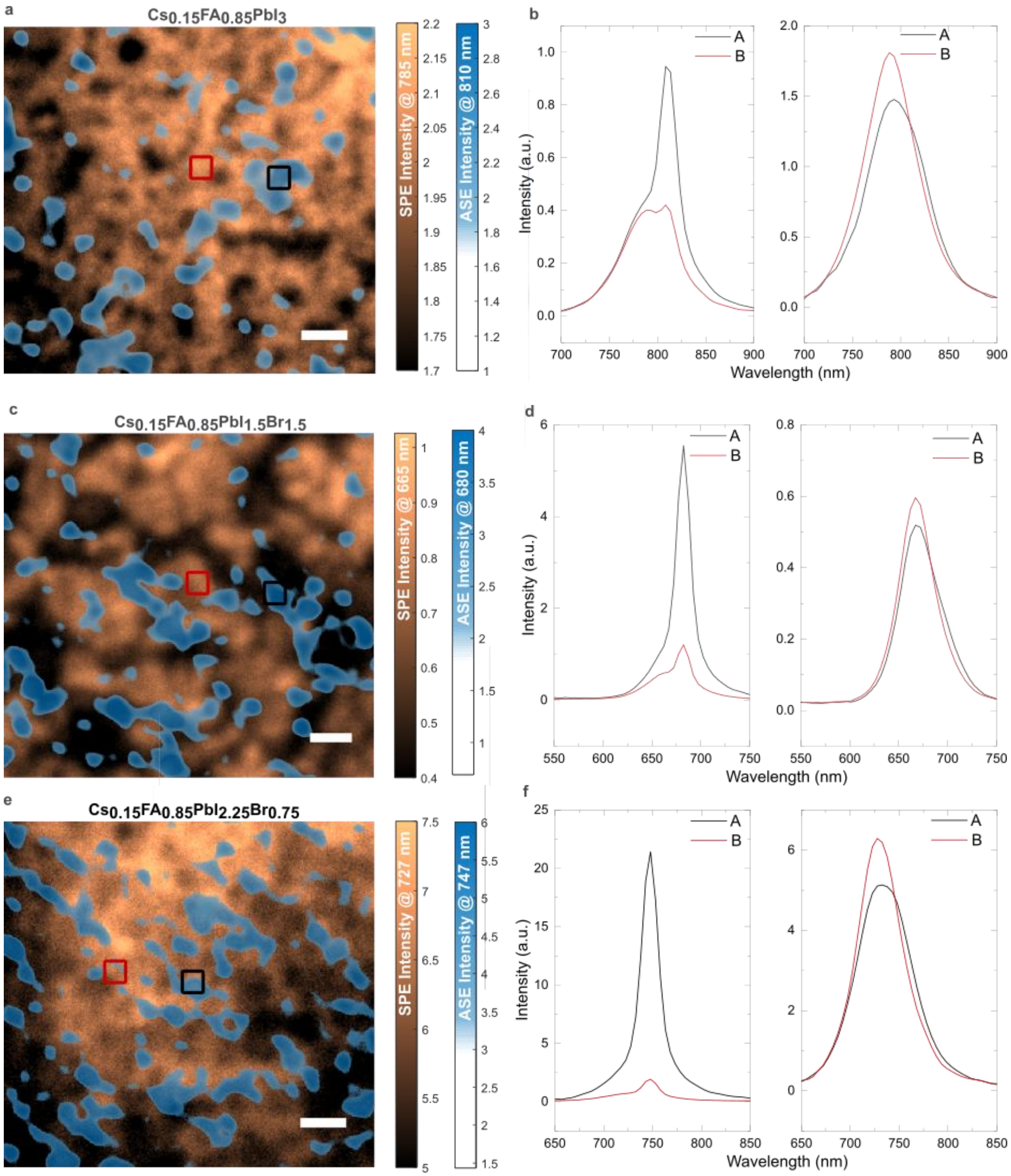

Figure. S6. Anti-correlation of ASE hotspot and SPE intensity in $Cs_{0.15}$ $FA_{0.85}PbI_xBr_{3-x}$, a,c,e: spatial overlap between ASE intensity map and SPE intensity map. **b,d,f:** (left) the ASE spectra, (right) the SPE intensity spectra. **(a,b)** x = 3**, (c,d)** x =1.5**, (e,f)** x=2.25**.** ROI A (black lines and boxes) represent the intense ASE hotspot, which shows a broader and lower SPE intensity. ROI B (red lines and boxes) represents the low ASE region but shows a narrower and higher SPE intensity. It is shown that such anti-correlation phenomenon can be observed

not only in $MAPbI_3$ samples, but also in FA-based samples. Such demonstration indicates that the phenomenon is a general phenomenon in 3D metal halide perovskite thin films.

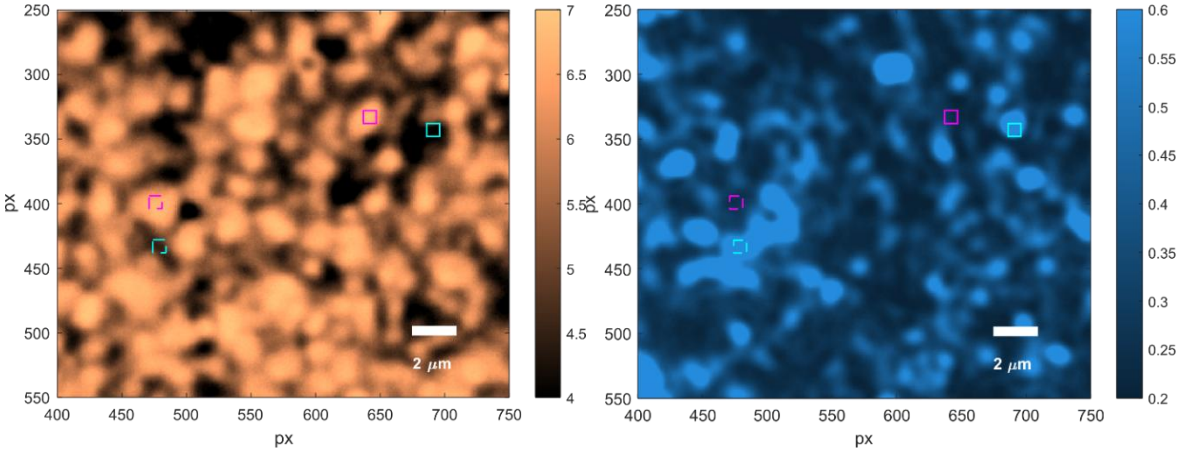

Figure. S7. Original figure of Figure 2b. left: Quasi-steady-state spontaneous emission map, measured below the threshold fluence ≈ 10 μJcm^{-2}. Right: ASE intensity maps, measured above the threshold fluence ≈ 112 μJcm^{-2}.

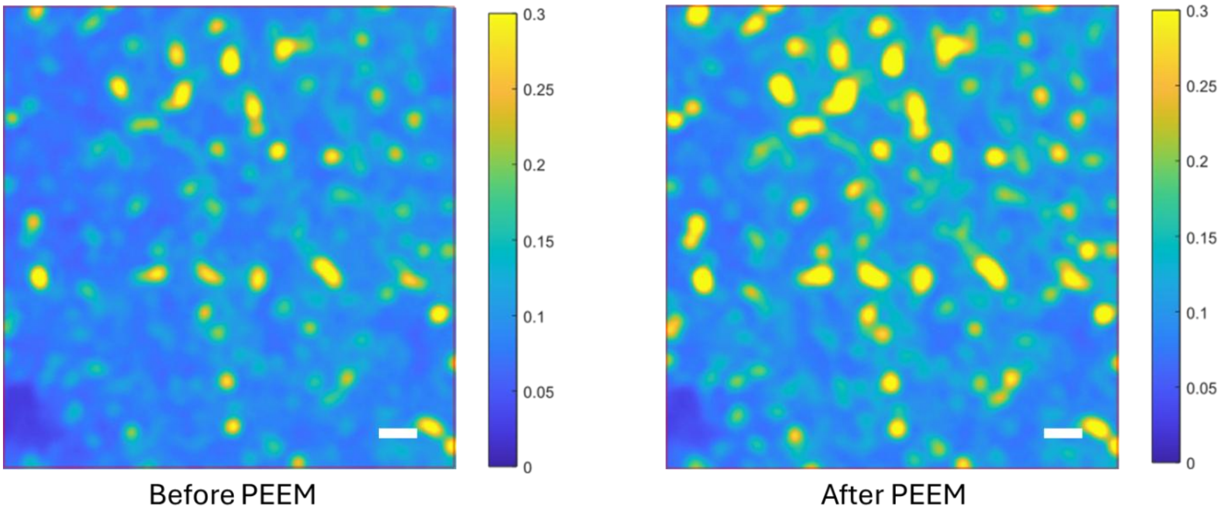

Figure. S8. PL emission map centered at 790 nm, taken before and after the PEEM measurements.

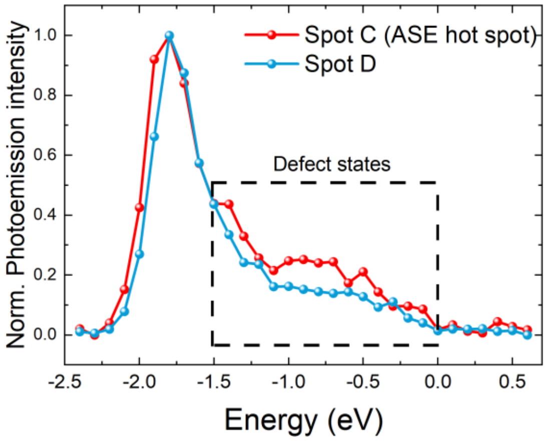

Figure. S9. The photoemission spectra of the spot C&D in Fig 3a of the main text.

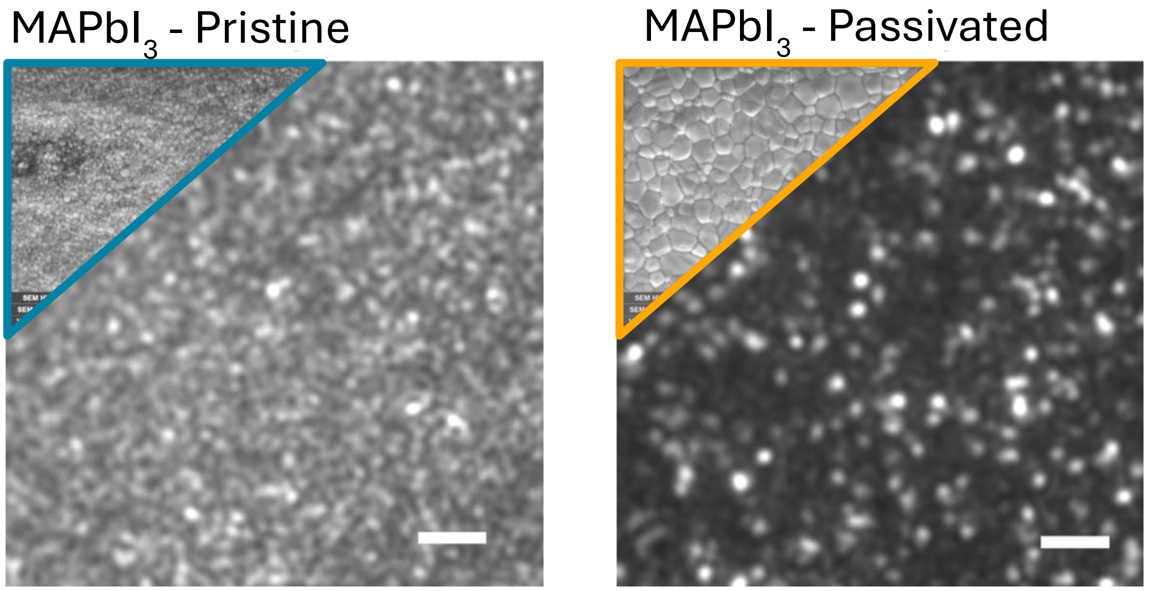

Figure. S10. PL images centered at 790 nm of a. pristine **b.** urea additive MAPbI3 thin film. The bright spots indicate the region that emits the ASE signals. The inset figure is the SEM image of the sample on the same scale. Scale bar = 2 µm, excitation fluence ~ 100 µJ cm^{-2}.
